# Genome-wide SNP data and morphology support the distinction of two new species of *Kovarikia* Soleglad, Fet & Graham, 2014 endemic to California (Scorpiones, Vaejovidae)

**DOI:** 10.3897/zookeys.739.20628

**Published:** 2018-02-22

**Authors:** Robert W. Bryson Jr., Dustin A. Wood, Matthew R. Graham, Michael E. Soleglad, John E. McCormack

**Affiliations:** 1 Department of Biology and Burke Museum of Natural History and Culture, University of Washington, Box 351800, Seattle, WA 98195-1800, USA; 2 Moore Laboratory of Zoology, Occidental College, 1600 Campus Road, Los Angeles, California 90041, USA; 3 U.S. Geological Survey, Western Ecological Research Center, San Diego Field Station, 4165 Spruance Road, Suite 200, San Diego, CA 92101, USA; 4 Department of Biology, Eastern Connecticut State University, 83 Windham Street, Willimantic, CT 06226, USA; 5 32255 Safflower St., Winchester, CA 92596, USA

**Keywords:** arachnid, Bayes factor delimitation, RADseq, species tree, Vaejovidae

## Abstract

Morphologically conserved taxa such as scorpions represent a challenge to delimit. We recently discovered populations of scorpions in the genus *Kovarikia* Soleglad, Fet & Graham, 2014 on two isolated mountain ranges in southern California. We generated genome-wide single nucleotide polymorphism data and used Bayes factors species delimitation to compare alternative species delimitation scenarios which variously placed scorpions from the two localities with geographically adjacent species or into separate lineages. We also estimated a time-calibrated phylogeny of *Kovarikia* and examined and compared the morphology of preserved specimens from across its distribution. Genetic results strongly support the distinction of two new lineages, which we describe and name here. Morphology among the species of *Kovarikia* was relatively conserved, despite deep genetic divergences, consistent with recent studies of stenotopic scorpions with limited vagility. Phylogeographic structure discovered in several previously described species also suggests additional cryptic species are probably present in the genus.

## Introduction

Species delimitation of morphologically conserved taxa has been a historically challenging endeavor for taxonomists. Recent developments in both DNA sequencing and species delimitation modeling have alleviated much of this burden by providing researchers with new ways to systematically classify similar-looking yet evolutionary distinct species. Although many species delimitation methods were originally designed and tested using only a handful of genes (e.g., Yang and Rannala 2010, Grummer et al. 2014), the rapid development of next-generation DNA sequencing means these methods can now be used with thousands of base-pairs of DNA acquired from across the entire genome (Leaché et al. 2014, Yang and Rannala 2014, Yang 2015).

Scorpions represent a well-known group of animals with a relatively conserved morphology. Thought to be derived from amphibious ancestors that lived more than 425 million years ago, their body plan appears to have changed relatively little since their adaptation to land (Coddington et al. 2004). Recent molecular studies have found that many wide-ranging scorpion species often represent species complexes (e.g., Bryson et al. 2013, 2016, Miller et al. 2014, Graham et al. 2017), with conserved external morphologies often masking more complex evolutionary histories. In these studies, geography was shown to be a better predictor of diversity than the morphological characters used to delineate species.

California is a global biodiversity hotspot and home to numerous endemic scorpions (i.e., Soleglad and Fet 2004, Soleglad et al. 2011, Webber et al. 2012, Savary and Bryson 2016). Scorpions in the genus *Kovarikia* Soleglad, Fet & Graham, 2014 are restricted to humid rocky microhabitats in several counties of southern California. The three currently recognized species in the genus, *K. angelena* (Gertsch & Soleglad, 1972), *K. bogerti* (Gertsch & Soleglad, 1972), and *K. williamsi* (Gertsch & Soleglad, 1972), are relatively rare and known from fewer than seven documented localities each (Soleglad et al. 2014). We discovered populations of *Kovarikia* on two different mountains seemingly isolated from other species in the genus. To test the hypothesis that they might represent new species, we generated genome-wide single nucleotide polymorphism data and used species delimitation modelling to compare alternative species delimitation scenarios which variously placed scorpions from the two new localities with geographically adjacent species or into separate lineages. We then estimated a time-calibrated phylogeny of *Kovarikia* and examined and compared the morphology of specimens from across its distribution. Using this approach, we address the taxonomy of *Kovarikia* and provide a key for species identification.

## Methods

### Taxon sampling, DNA sequencing, and SNP data assembly

We sequenced 36 samples of *Kovarikia* from 16 localities representing all described species and the new populations from the Santa Ana and San Gabriel Mountains (Table 1). We generated genome-wide single nucleotide polymorphism (SNP) data using the double-digest restriction-digest associated DNA marker (ddRADseq) protocol of Peterson et al. (2012). We extracted high molecular-weight genomic DNA from pedipalp tissue using Qiagen DNeasy Blood & Tissue Kits (Qiagen Inc.), and followed the ddRADseq wet-lab protocol for scorpions published in Bryson et al. (2016). Pooled ddRAD libraries were sent to the Vincent J. Coates Genomics Sequencing Laboratory at UC Berkeley for 150-base single-end sequencing on one lane of an Illumina HiSeq4000 (combined with 10 pooled libraries in the lane).

**Table 1.** Genetic samples of *Kovarikia* used in this study. Additional details on collecting localities are listed in Appendix 1.

Sample numbers	Species	Locality
sky241, sky263, sky264	*K. angelena*	CA: Ventura Co: Yerba Buena Road, Santa Monica Mountains
sky464, sky465, sky466, sky499	*K. angelena*	CA: Los Angeles Co: Kanan-Duma Road, Santa Monica Mountains
sky266, sky498	*K. bogerti*	CA: San Bernardino Co: Mountain Home, San Bernardino Mountains
sky470, sky471, sky472	*K. bogerti*	CA: Riverside Co: Mountain Center, San Jacinto Mountains
sky467, sky468, sky469	San Gabriel Mtns	CA: Los Angeles Co: Eaton Canyon Falls, San Gabriel Mountains
sky250, sky516, sky517	Santa Ana Mtns	CA: Orange Co: Trabuco Creek Road, Santa Ana Mountains
sky518	Santa Ana Mtns	CA: Orange Co: Silverado Canyon Road, Santa Ana Mountains
sky248, sky249	*K. williamsi*	CA: San Diego Co: Palomar Mountain
sky251	*K. williamsi*	CA: San Diego Co: San Diego Zoo Safari Park
sky274, sky276	*K. williamsi*	CA: San Diego Co: Barrett Flume
sky277	*K. williamsi*	CA: San Diego Co: Barrett Lake Road
sky275, sky519, sky520	*K. williamsi*	CA: San Diego Co: Escondido
sky511	*K. williamsi*	CA: San Diego Co: Indian Valley Road
sky273, sky504	*K. williamsi*	CA: San Diego Co: Mission Trails
sky512, sky513	*K. williamsi*	CA: San Diego Co: Padre Dam
sky494, sky514, sky515	*K. williamsi*	CA: San Diego Co: Santa Ysabel

We demultiplexed and processed Illumina reads using pyRAD v2.16.1 (Eaton and Ree 2013, Eaton 2014). Sequences were clustered at 90% similarity within samples using USEARCH v7.0.1090 (Edgar 2010) and aligned with MUSCLE v3.8.31 (Edgar 2004). Error rate and heterozygosity were jointly estimated from the base counts in each site across all clusters. Consensus sequences with less than five reads, more than five undetermined sites, more than five heterozygous sites, or more than two haplotypes were discarded. Consensus sequences were then clustered across samples using the same 90 % similarity threshold and aligned. Any locus with a site appearing heterozygous across more than 50 % of samples was discarded as likely representing a clustering of paralogs. We set the minimum number of samples in a final locus to 16, allowing up to 46 % missing data per locus.

### Species delimitation

**Figure 1.**
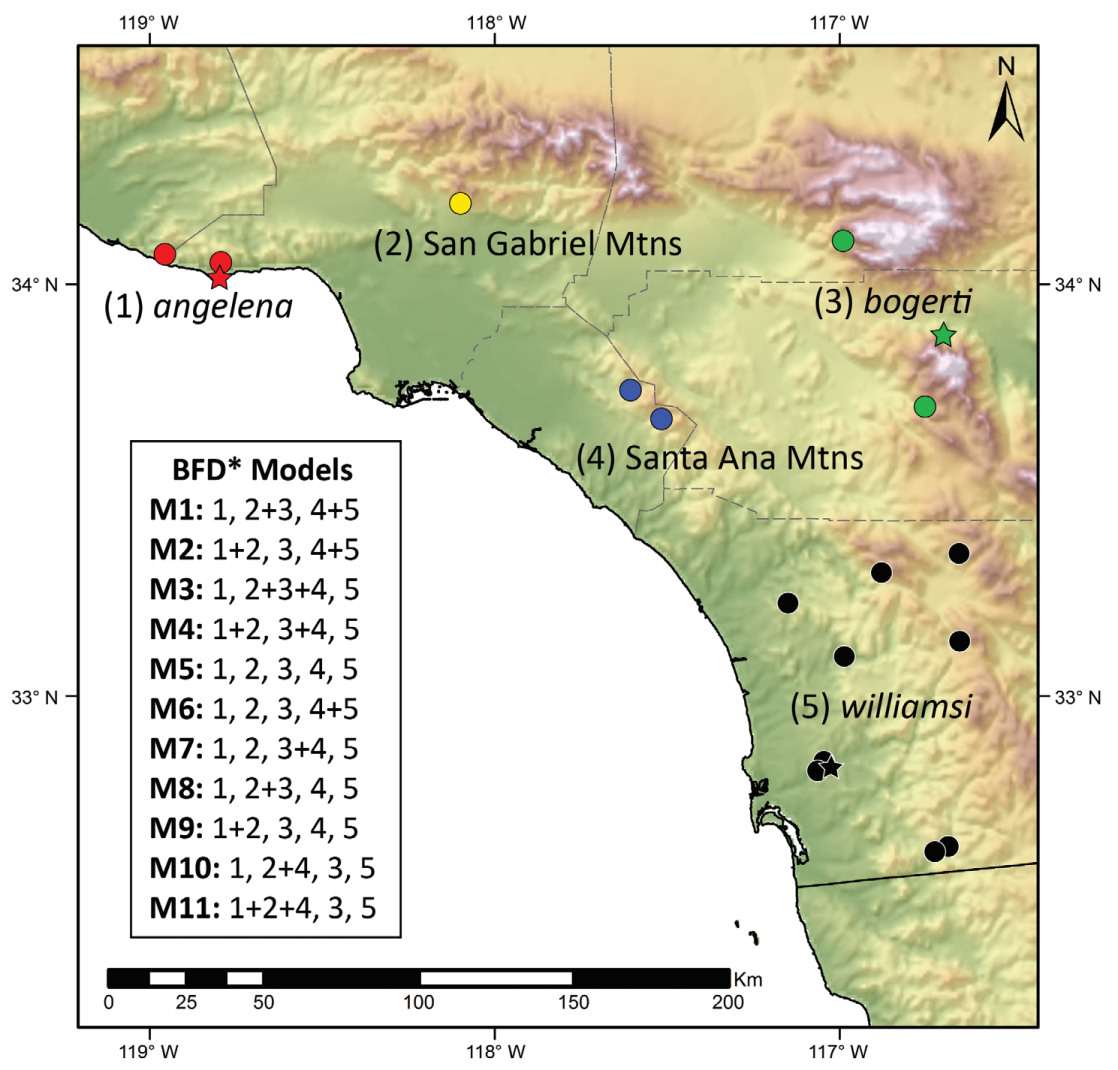
Sampling localities for genetic samples of scorpions in the genus *Kovarikia*. Type localities (star symbols) are shown for reference. Inset shows the 11 competing BFD* models used in species delimitation; numbers correspond to species (**1** *K. angelena* **3** *K. bogerti* **5** *K. williamsi*) or new localities (**2** San Gabriel Mountains **4** Santa Ana Mountains).

We performed Bayes factor species delimitation using BFD* (Grummer et al. 2014, Leaché et al. 2014) implemented using the SNAPP v1.3.0 (Bryant et al. 2012) plugin for BEAST v2.4.3 (Bouckaert et al. 2014). We tested 11 competing models which variously placed scorpions from the two new localities with geographically adjacent species or into separate lineages (Fig. 1, Table 2). We set the unsampled mutation rates *u* and *v* to 1, alpha to 1, beta to 250, lambda to 20, sampled a coalescence rate initially set to 10, and used default settings for all other parameters. We conducted path sampling for a total of 24 steps, running each for 200,000 MCMC generations and sampling every 1,000 steps to estimate marginal likelihoods for each competing model. We ranked and compared the resulting marginal likelihood values using Bayes factors (Kass and Raftery 1995). We repeated the analyses using default settings for the mutation rates u and v, alpha, beta, lambda, and coalescence rate to evaluate potential impacts of using different priors.

**Table 2.** Bayes factor comparisons of 11 competing models of species delimitation in *Kovarikia*. Marginal likelihood estimates (MLE) and Bayes factors comparisons (2*ln*BF) shown; the model that received the best marginal likelihood score is indicated by a 2*ln*BF score of NA. SGM = San Gabriel Mountains, SAM = Santa Ana Mountains.

Model	Species	Groupings	MLE	Rank	2lnBF
M1	3	*angelena*, SGM + *bogerti*, SAM + *williamsi*	-25035.20	8	14165.49
M2	3	*angelena* + SGM, *bogerti*, SAM + *williamsi*	-26835.35	9	15965.64
M3	3	*angelena*, SGM + *bogerti* + SAM, *williamsi*	-24961.18	7	14091.47
M4	3	*angelena* + SGM, *bogerti* + SAM, *williamsi*	-28211.23	11	17341.52
M5	5	*angelena*, SGM, *bogerti*, SAM, *williamsi*	-10869.71	1	–
M6	4	*angelena*, SGM, *bogerti*, SAM + *williamsi*	-14205.08	2	3335.37
M7	4	*angelena*, SGM, *bogerti* + SAM, *williamsi*	-14849.95	3	3980.24
M8	4	*angelena*, SGM + *bogerti*, SAM, *williamsi*	-18460.44	4	7590.73
M9	4	*angelena* + SGM, *bogerti*, SAM, *williamsi*	-19812.85	6	8943.14
M10	4	*angelena*, SGM + SAM, *bogerti*, *williamsi*	-19084.64	5	8214.93
M11	3	*angelena* + SGM + SAM, *bogerti*, *williamsi*	-27947.41	10	17077.70

We generated a final species tree in SNAPP based on the best-ranked species model from BFD*. We ran the analysis for 1,000,000 MCMC generations, sampling every 1,000 steps. We confirmed convergence and high ESS values using Tracer v1.5 (Rambaut and Drummond 2009), and produced a maximum clade credibility tree after a 25 % burnin using TreeAnnotator v1.7.5 (Drummond and Rambaut 2007).

### Phylogenetic relationships

To examine phylogenetic relationships within *Kovarikia* and estimate approximate dates of divergences among lineages, we estimated a time-calibrated phylogeny from the concatenated RAD loci using BEAST v1.8.2 (Drummond et al. 2012). We used the same priors and calibrations specified in Bryson et al. (2016), which included giving the ucld.mean parameter a lognormal distribution to span mean substitution rates previously calculated for nine nuclear genes in scorpions (Gantenbein and Keightley 2004). We ran the analysis for 80 million generations and retained trees and parameters every 10,000 steps. We displayed results in Tracer to assess convergence and effective sample sizes for all estimated parameters. We discarded the first 25% of trees as burnin and summarized the maximum clade credibility (MCC) tree with median heights using TreeAnnotator v1.8.2 (Drummond et al. 2012). We repeated the analysis using different starting seeds to confirm adequate mixing and consistent results.

### Morphological assessments

We examined the morphology of 40 preserved specimens of *Kovarikia* (Appendix 1). Our terminology and conventions followed Stahnke (1971) and Sissom et al. (1990) for mensuration, Soleglad and Sissom (2001) for pedipalp finger dentition and chelal carinae, Vachon (1974) for trichobothrial patterns, Soleglad and Fet (2003a) for sternum terminology, Soleglad and Fet (2003b) for cheliceral dentition terminology, and Soleglad and Fet (2008) and Ayrey and Soleglad (2015) for the hemispermatophore description. We limited the use of morphological data published in previous studies to avoid possible researcher-based biases in counts or measurements.

## Results

### Genetic data

One sample of *K. williamsi* from the San Diego Zoo Safari Park contained a high percentage of missing data (>90 %) and was not included in the final SNP data assembly. The final aligned data set with all RAD loci contained 35 samples, 2,915 loci and 414,566 nucleotides. The final data set for species delimitation contained 35 samples and 1,123 unlinked SNPs after sites with missing data were removed by SNAPP. Datasets were deposited in Dryad.

### Species delimitation

**Figure 2.**
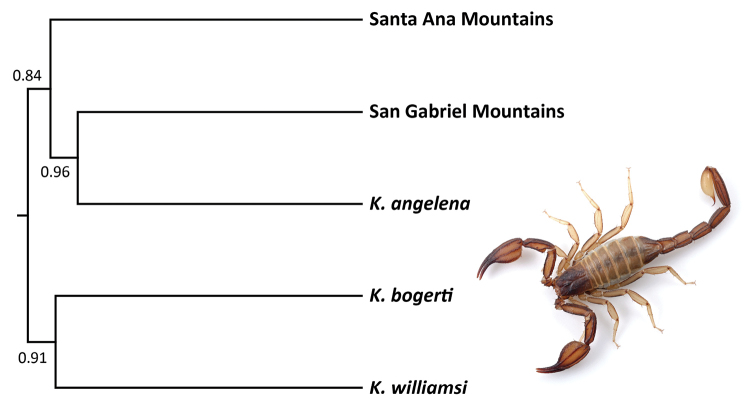
Species tree of scorpions in the genus *Kovarikia* reconstructed from 1,123 unlinked SNPs, based on the best-ranked five-species model (Table 2). Numbers represent posterior probability support for nodes. Shown is an adult female *Kovarikia* from the San Gabriel Mountains.

The BFD* model with the best marginal likelihood value strongly supported a five-species model that placed scorpions from the San Gabriel and Santa Ana Mountains into separate lineages (Table 2). Analyses using default settings resulted in the same model rankings. The MCC species tree showed uncertainty in the phylogenetic placement of these five species (Fig. 2). *Kovarikia angelena* and scorpions from the San Gabriel Mountains were placed together in a strongly supported relationship. Scorpions from the Santa Ana Mountains were sister to this grouping, although nodal support for this relationship was weak (0.84 posterior probability). *Kovarikia bogerti* and *K. williamsi* were placed together in a separate clade with 0.91 posterior probability support.

### Phylogenetic relationships

**Figure 3.**
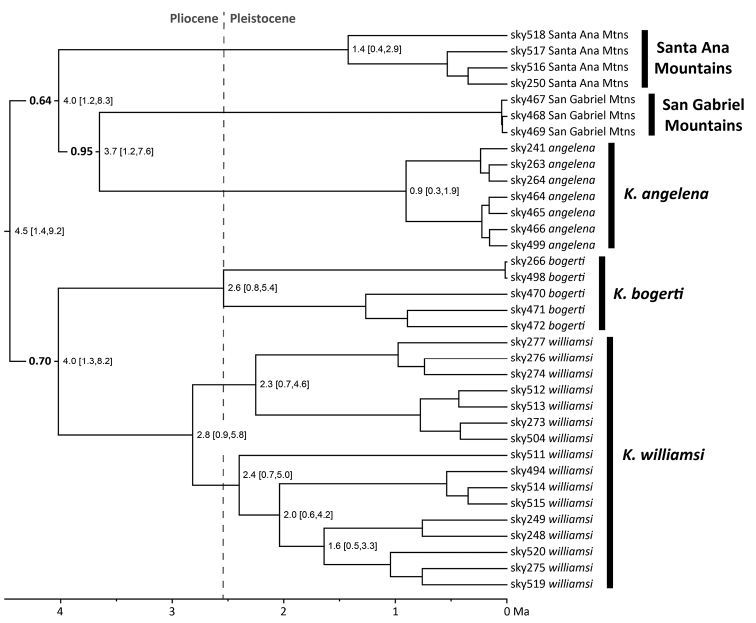
Time-calibrated phylogeny of scorpions in the genus *Kovarikia* inferred from 414,566 base-pairs of concatenated RAD loci. Nodes that received less than 1.0 posterior probability support are labeled and in bold. Mean estimated divergence date (in millions of years ago, Ma) followed by 95% highest posterior density intervals in brackets. SBM = San Bernardino Mountains, SJM = San Jacinto Mountains.

Phylogenetic analysis of the concatenated RAD loci produced a well-supported tree (Fig. 3). Samples from the San Gabriel Mountains, the Santa Ana Mountains, and *K. angelena*, *K. bogerti*, and *K. williamsi* formed five unique clades. The relationships among these clades matched those in the species tree (Fig. 2), although two nodes near the base of the tree were not strongly supported (< 0.95 posterior probability). Phylogeographic structure was present within *K. williamsi*, with samples from the northern distribution (Escondido, Palomar Mountain, Indian Valley Road, and Santa Ysabel) and southern distribution (Mission Trails, Padre Dam, Barrett Flume, and Barrett Lake Road) forming two well-supported clades (“North” and “South”, Fig. 3). Additional phylogeographic structure was present in each of these clades of *K. williamsi*. *Kovarikia bogerti* from the San Bernardino Mountains and San Jacinto Mountains also formed geographically cohesive clades (Fig. 3).

Estimated divergence dates among the five major clades of *Kovarikia* predated the start of the Pleistocene 2.6 million years ago (Ma), based on mean dates (Fig. 3). However, 95% posterior credibility intervals for divergence date estimates were large, encompassing millions of years. Mean dates of divergences among clades within *K. williamsi* and *K. bogerti* were estimated between 1.6–2.8 Ma.

### Morphological assessments

**Figure 4.**
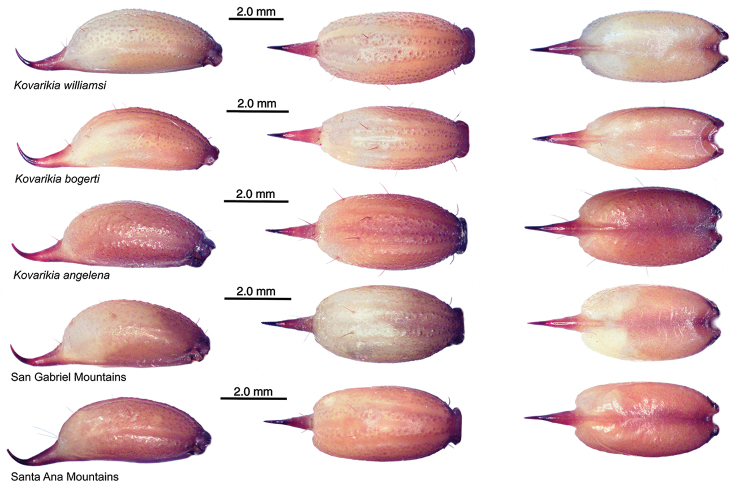
Telson of adult females of genus *Kovarikia*, lateral (left column), ventral (middle column), and dorsal (right column) views. In *K. bogerti* the vesicle width is noticeably thinner than in the other species and the vesicular ridges are more reduced (see ventral and dorsal views). Note, the vesicular linear patch found on the dorsal surface of adult males is absent in the females.

Morphological assessments revealed several characters that differentiated *K. angelena*, *K. bogerti*, *K. williamsi*, and specimens from the San Gabriel Mountains and the Santa Ana Mountains (Appendix 2). Telson shape varied substantially among and within the species, with female telsons of *K. bogerti* and *K. williamsi* being smaller than those of *K. angelena*, specimens from the San Gabriel Mountains, and specimens from the Santa Ana Mountains (Fig. 4). Female telsons of *K. bogerti* were significantly less wide than those of *K. williamsi*. *Kovarikia angelena* and specimens from the Santa Ana Mountains exhibited relatively longer telson vesicles than *K. bogerti*, *K. williamsi*, and specimens from the San Gabriel Mountains. Both fixed (FF) and movable (MF) chelal fingers in female *K. bogerti* and *K. williamsi* were also longer than those of *K. angelena*, specimens from the San Gabriel Mountains, and specimens from the Santa Ana Mountains. Carapace lengths varied, with *K. williamsi* being the largest species and *K. angelena* being the smallest. Pectine tooth counts overlapped in both sexes for all species except *K. angelena*, which had fewer pectine teeth. Pectine tooth counts were highest for the largest species, *K. williamsi*, and lowest for the smallest species, *K. angelena*. Additional details on morphological characters are provided in the Discussion. These differences were used to diagnose five species in our “Key to Species of *Kovarikia*”.

## Discussion

Our genetic data strongly support the recognition of *Kovarikia* from the San Gabriel Mountains and the Santa Ana Mountains as distinct lineages, which we describe as new species and name below. Based on the topography of southern California and our current understanding of the distribution of *Kovarikia* (Fig. 1), both species appear to be geographically isolated. Morphology among the species of *Kovarikia* is relatively conserved, despite deep genetic divergences, consistent with recent studies of other stenotopic scorpions (e.g., Bryson et al. 2013; Talal et al. 2015). Phylogeographic structure within *K. williamsi* and *K. bogerti* suggest additional cryptic species are probably present in the genus and reveal the need for future research with denser sampling. Although California is a relatively well-studied state biologically, our study highlights the fact that new populations await discovery and that morphologically conserved taxa such as scorpions, and other arachnids (e.g., Satler et al. 2013), likely harbor unexpected species-level diversity.

## Systematics

### Family Vaejovidae Thorell, 1876

#### Subfamily Vaejovinae Thorell, 1876

##### Genus *Kovarikia* Soleglad, Fet & Graham, 2014

###### Kovarikia savaryi

Taxon classificationAnimaliaORDOFAMILIA

Bryson, Graham & Soleglad sp. n.

http://zoobank.org/CB431CD7-8707-43FC-9152-79D4FDE90428

**Figure 5.**
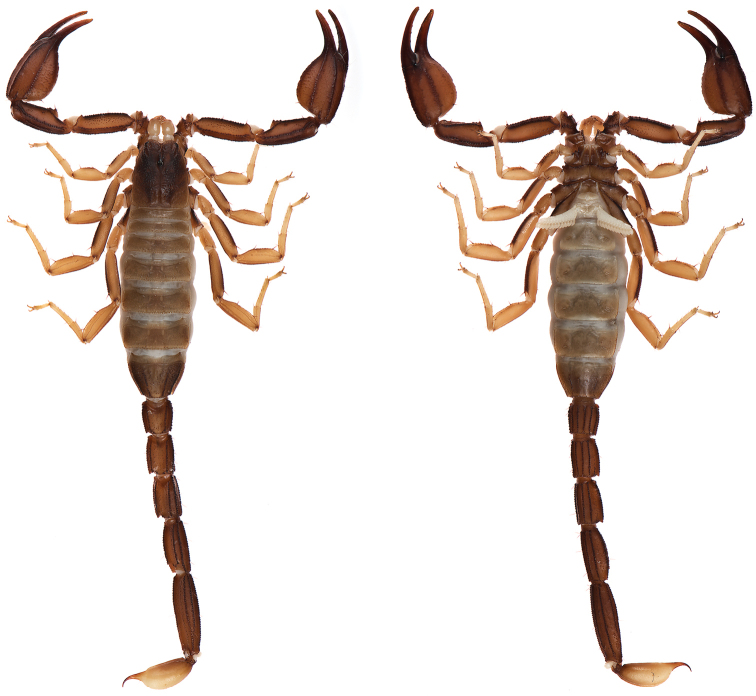
Dorsal and ventral views of *Kovarikia savaryi* sp. n. male holotype.

**Figure 6.**
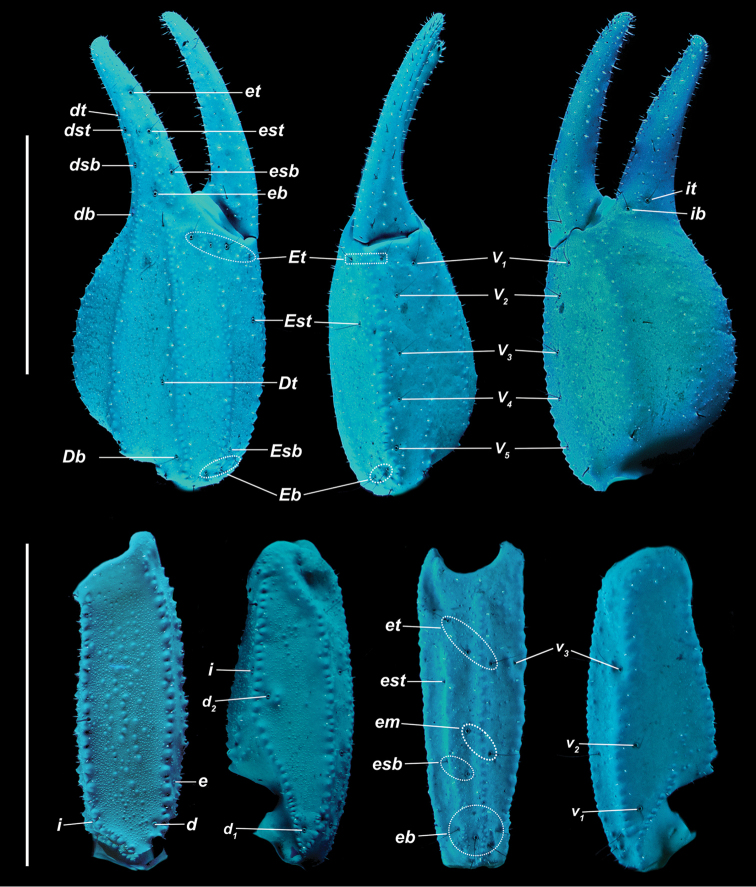
*Kovarikia savaryi* sp. n. male holotype. Trichobothrial pattern. Scale bar = 5 mm; top bar for chela, bottom applies to femur and patella.

**Figure 7.**
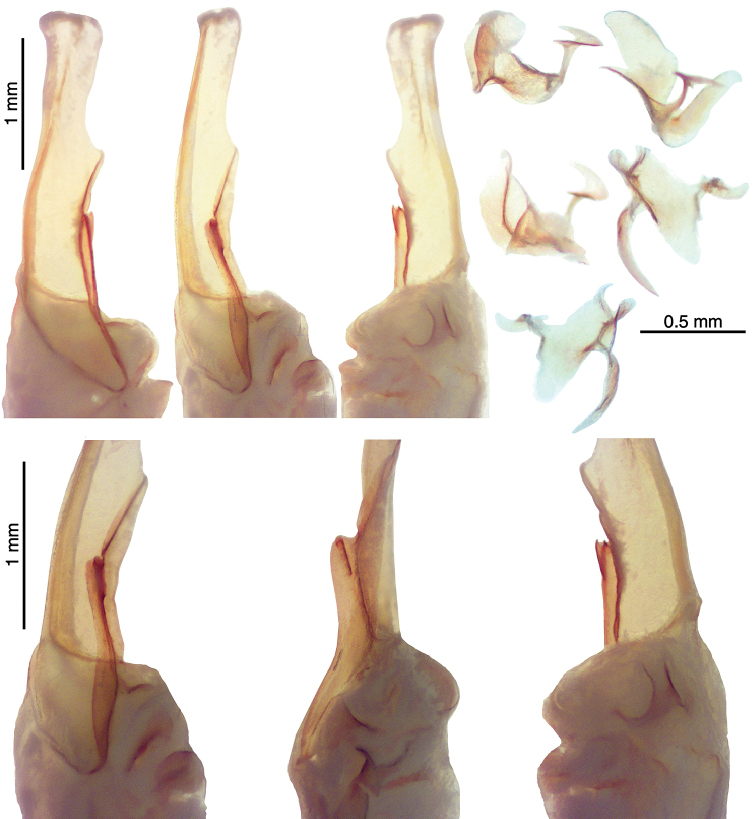
*Kovarikia savaryi* sp. n., male holotype. Right hemispermatophore and mating plug (submerged in alcohol). **Upper-Left** Hemispermatophore median area and lamina, dorsal, interodorsal, and ventral views **Lower** Closeup of the median area and lamellar hooks, interodorsal, internal, and ventral views. Note, embedded mating plug is visible in ventral view **Upper-Right** Mating plug, four dorsal views at various angles and one exteroventral view (bottom).

Figs 5 , 6 , 7 ; Table 3

####### Type material.

*United States*: *California*: *Orange Co*: male holotype (DMNS ZA.38170), Trabuco Creek Road near the entrance to Holy Jim Canyon, Santa Ana Mountains. 33.67699°N, 117.51733°W, 527 m. 15 April 2015. R.W. Bryson. Paratypes: Same locality. 15 April 2015. R.W. Bryson. 1 ♂, 5 ♀ (DMNS ZA.38171–ZA.38176). *Orange Co*: Silverado Canyon Road, Santa Ana Mountains. 33.74614, -117.59327, 524 m. 16 April 2015, R.W. Bryson. 1 ♂ (DMNS ZA.38177).

####### Etymology.

Patronym honoring Warren E. Savary for his contributions to vaejovid scorpion taxonomy.

####### Diagnosis.

Large sized species for the family, with males up to 50.5 mm and females reaching 57.0 mm; pectinal tooth counts 12–13 for males and 11–13 for females. The species possesses the characteristics of genus *Kovarikia*: i.e. neobothriotaxy on ventral surface of chela, secondary lamellar hook on spermatophore, large crescent-shaped barb with a smooth edge on the mating plug, and secondary exteromedian (*EM_c_*) carina on pedipalp patella (Soleglad et al. 2014). The holotype differs from the *K. oxy* sp. n. holotype in the following: median eyes protrude only slightly above carapace surface (eyes are well above in *K. oxy*); median carinal pair on sternite VII essentially obsolete except for a few scattered small granules (obsolete in *K. oxy*); moderately granular intermediary carinae occur on metasomal segment I, the posterior 1/5 of segment II, and posterior 1/6 of segment III (strongly granular on segment I, the posterior 1/4 on segment II and posterior 1/5 of segment III in *K. oxy*); lateral carinae on metasomal segment V crenulate and connecting with dorsolateral carinae at posterior 1/3 of segment (posterior 1/4 in *K. oxy*); internal surface of femur with scattered granules of various size, mostly on proximal 1/2 (few large granules arranged in a line along proximal 1/3 in *K. oxy*); basitarsus retroventral setae count of 4/4:5/5:5/5:7/6 (4/4:7/7:7/7:8/7 in *K. oxy*). Differs from the other *Kovarikia* spp. by pectine counts and morphology of the chelal fingers and telson, as outlined below in the “Key to Species of *Kovarikia*”.

####### Description of holotype.

*Color* (Fig. 5): Carapace, trochanter, femur, patella, tergites, and metasoma have a brown base color with dark brown to black markings along the carinae of the pedipalp and metasoma. Legs are yellow brown with dark brown carinae. Pedipalp chelae are brown in color with darker reddish-brown coloration at the anterior portion of the palm where the fixed finger and movable finger meet. Chelicerae are light yellow with dark reddish-brown dentition. Vesicle portion of the telson is yellow-orange proximally, fading to very light yellow on the distal third, with a dark reddish-brown to black aculeus. Pectines and genital operculum are light yellow to cream colored. *Morphology*: Carapace: trapezoidal with strongly emarginated anterior margin; surface with scattered granules; a strong median furrow traverses length of carapace; ratio of location of median eyes location (from anterior edge)/carapace length = 0.348; median eyes protrude only slightly above carapace surface. Tergites: surface with small granules on distal 1/3–2/3 of tergites II–VI; tergite VII with two pairs of granular lateral carinae, and a strong median hump. Sternites: III–VI smooth to very finely granular and without carinae; VII with granular ventral lateral carinae on posterior 2/3, median carinal pair essentially obsolete except for a few scattered small granules. Spiracles: slightly ellipsoid and with median side rotated 30° away from posterior sternite margin. Genital Operculum: sclerites separated on posterior 1/5 exposing conspicuous genital papillae. Pectines: tooth count 12/13; middle lamellae 7/6; sensorial areas present on all pectine teeth. Metasoma: ratio of segment I length/width 1.15; segment II length/width 1.39; segment III length/width 1.56; segment IV length/width 2.09; segment V length/width 3.33. Segments I–IV: dorsal carinae are moderately denticulate on segments I–IV and have slightly enlarged distal denticles; dorsolateral carinae are moderately denticulate on segments I–IV with slightly enlarged posterior denticles; ventrolateral carinae are moderately crenulate on segments I–IV; moderately granular intermediary carinae occur on segment I, the posterior 1/5 of segment II, and posterior 1/6 of segment III; ventromedian carinae are crenulate on segments I–IV; ventrolateral setae 2/2:2/2:2/2:2/2; ventral submedian setae 2/2:3/3:3/3:3/3. Segment V: dorsolateral carinae crenulate; lateral carinae crenulate and connecting with dorsolateral carinae at posterior 1/3 of segment; ventrolateral carinae crenulate; ventromedian carinae crenulate; intercarinal spaces with sparsely scattered granules; dorsolateral setation 2/2; lateral setation 2/2; ventrolateral setation 4/4; ventromedian setation 4/4. Telson: smooth to slightly granular with no subaculear tubercule and lacking laterobasal aculear serrations (LAS; Fet et al. 2006); posterior end of vesicle inflated toward the aculeus forming a pair of smooth ventral grooves and carinae that stop at aculeus base; vesicle length/width 2.00; vesicle length/depth 2.54. Chelicerae: dorsal edge of fixed finger with four teeth, one distal, one subdistal, one median, and one basal, the latter two denticles formed as a bicuspid; ventral edge smooth; dorsal edge of movable finger has five teeth total comprised of one distal, two subdistal, one median tooth, and one basal tooth; ventral edge smooth; serrula with approximately 25 tines. Pedipalps (Fig. 6): trichobothrial pattern type C, neobothriotaxic: trichobothria *ib-it* positioned on very base of fixed finger, distance between positions of *Dt* and *Est* is less than that of *Dt* from palm base, *Db* dorsal of digital carina, *Et1* is clearly closer to the movable finger than V1, five ventral trichobothria (*V1*–*V5*); ratio of chela length/width 3.33; femur length/width 2.84; patella length/width 2.33; fixed finger length/carapace length 0.66. Chela: median denticles (MD) of fixed finger aligned and divided into six subrows by five outer denticles (OD); flanked by six inner denticles (ID); movable finger with six subrows of MD, five OD and seven ID. Chela carinae: Digital carina strong and crenulate; subdigital carina strong and crenulate but obsolete on distal 7/8; dorsosecondary moderate and crenulate; dorsomarginal very rounded, with large scattered granules; dorsointernal obsolete; interomedian weak, rounded with scattered granules; external carina moderate and crenulate; ventroexternal strong and crenulate; ventromedian moderate and crenulate on proximal 1/5, fading to scattered granules and essentially obsolete on distal 3/5; ventrointernal moderate to weak, rounded, with small scattered granules. Femur: dorsointernal and ventrointernal strong, black in color, and crenate; dorsoexternal carinae crenulate, brown to burgundy in color; ventroexternal reddish orange with scattered granules of various size; internal surface has scattered granules of various size, mostly on proximal 1/2. Patella: dorsointernal and dorsoexternal carinae are crenulate and dark brown to burgundy in color; ventral internal and ventroexternal carinae crenate to crenulate and dark brown to burgundy; external median carinae dark brown to burgundy and crenulate; seconday external median carina strong and on proximal 3/4, obsolete on distal 1/4, dark brown to burgundy and crenulate; internal surface has a large spur flanked by a few large granules. Legs: Ventral surface of telotarsi with single median row of 17–27 spinules terminating distally with two pairs of spinules. Two rows of small spinules occur on all basitarsi, fading proximally, but are very weak on basitarsus IV. Basitarsus populated with large irregularly placed darkly pigmented setae as follows: vental setae 5/5:5/6:6/6:6/5, retroventral setae 4/4:5/5:5/5:7/6, retromedian accessory setae 2/2:3/2:3/3:3/3, retrosuperior setae 2/2:2/2:2/2:2/2, and superior setae 3/3:3/3:3/3:3/3 (excluding DSM and DPS). Hemispermatophore (Fig. 7): Left hemispermatophore is 5.7 mm in length; lamina length 2.9, primary lamellar hook length 1.3, secondary lamellar hook length 0.5 (distance between tips of primary and secondary hooks), and trough difference (vertical distance between ventral and dorsal troughs) 1.0. Lamellar edges roughly parallel, a slight constriction adjacent to (distal) the secondary lamellar hook; terminus blunted with a very slight distal crest on the dorsal side. Primary lamellar hook extends somewhat from lamina base, is distinctly bifurcated, and is formed entirely from the dorsal trough. Secondary lamellar hook not bifurcated and forms a slight expansion of the lamina. A sclerotized mating plug with large asymmetric barb with a smooth edge was extracted from the ventrointernal aspect of the hemispermatophore median area.

**Table 3.** Measurements (in mm) of the type series of *Kovarikia savaryi* sp. n.

	**Male holotype (DMNS ZA.38170)**	**Male paratype 1 (DMNS ZA.38171)**	**Male paratype 2 (DMNS ZA.38177)**	**Female paratype 1 (DMNS ZA.38172)**	**Female paratype 2 (DMNS ZA.38173)**	**Female paratype 3 (DMNS ZA.38174)**	**Female paratype 4 (DMNS ZA.38175)**	**Female paratype 5 (DMNS ZA.38176)**
Total L	50	50.5	49	47.5	57	53	47	57
Cara L	5.85	6.5	6.15	5.75	6.5	6.3	6.05	7.2
Meso L	14.95	15.7	14.1	15.8	17.65	17.8	13.6	17.3
Met I L	3	2.95	2.9	2.75	3.1	2.95	2.65	3.2
Met I W	2.6	2.95	2.9	2.55	2.7	2.7	2.6	2.95
Met II L	3.55	3.55	3.5	3.25	3.65	3.5	3.3	3.9
Met II W	2.55	2.9	2.8	2.5	2.55	2.55	4.45	2.85
Met III L	3.9	3.95	3.9	3.55	3.9	3.75	3.5	4.2
Met III W	2.5	2.85	2.7	2.4	2.45	2.5	2.3	2.8
Met IV L	4.8	4.95	5	4.5	4.95	4.75	4.35	4.5
Met IV W	2.3	2.65	2.5	2.3	2.2	2.3	2.2	2.6
Met V L	7.5	7.3	7.65	6.85	7.7	7.35	6.7	8.2
Met V W	2.25	2.5	2.4	2.2	2.3	2.3	2.15	2.5
Tel L	-	6.3	-	6.2	7.2	6.9	6.2	7.3
Ves L	5.2	4.75	4.1	4.5	5.4	5.1	4.6	5.55
Ves W	2.6	2.6	2.8	2.35	2.6	2.5	2.5	3
Ves D	2.05	2.1	2.2	2	2.1	2.15	1.95	2.3
Acu L	-	1.6	1.6	1.7	1.85	1.75	1.65	1.7
Fem L	5.4	5.5	5.6	5.5	5.95	5.7	5.35	6.4
Fem W	1.9	1.95	1.9	1.8	2.1	2	1.95	2.2
Pat L	5.25	5.3	5.5	5.4	5.8	5.6	5.3	6.3
Pat W	2.25	2.25	2.35	2.3	2.3	2.35	2.3	2.6
Chel L	10	9.95	10.4	10.1	11.5	10.8	9.85	12.35
Palm L	5.55	5.8	5.9	5.55	6.4	6.05	5.5	6.8
Palm W	3	2.55	3.3	2	3.2	3.05	2.85	3.5
Palm D	4.2	4.45	4.65	2.8	4.3	3.95	4	4.75
FF L	3.85	5.2	4.1	4	4.4	4.1	3.75	4.75
MF L	5.25	5.15	5.5	5.25	6	5.55	5.05	6.45
Pect Teeth	12/13	13/12	12/12	11/11	12/11	11/10	11/11	12/13

####### Measurements of male holotype

**(mm).** Total L, 50.0; carapace L, 5.85; mesosoma L, 14.95; metasoma L (additive without telson), 22.75. Metasomal segments: I L/W, 3.00/2.60; II L/W, 3.55/2.55; III L/W, 3.90/2.50; IV L/W, 4.80/2.30; V L/W, 7.50/2.25. Telson: vesicle L/W/D, 5.20/2.06/2.05. Pedipalps: femur L/W, 5.40/1.90; patella L/W, 5.25/2.25; chela L/W/D, 10.00/3.00/4.20; fixed finger L, 3.85; movable finger L, 5.25; palm L, 5.55. Note: Aculeus is broken so Telson L and Aculeus L are omitted.

####### Male and female variability.

Slight sexual dimorphism was evident in telson and metasoma morphology for *K. savaryi*. Two-tailed Student’s t-tests indicated that the length of metasomal segment V is significantly larger in males (*p* = 0.048). The telson aculeus is significantly longer in females (*p*=0.019). Differences may also occur in lengths and widths of additional metasomal segments, as well as femur, patella, and chela morphology, but small sample sizes hindered statistical power in our analyses.

###### Kovarikia oxy

Taxon classificationAnimaliaORDOFAMILIA

Bryson, Graham & Soleglad sp. n.

http://zoobank.org/C9589607-5BA9-4986-A038-258A4E182CD6

**Figure 8.**
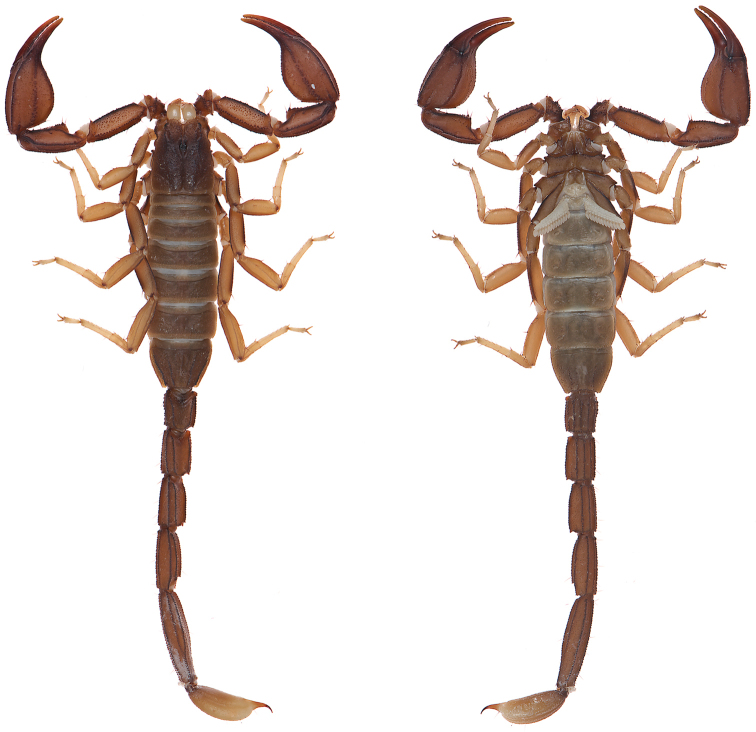
Dorsal and ventral views of *Kovarikia oxy* sp. n. male holotype.

**Figure 9.**
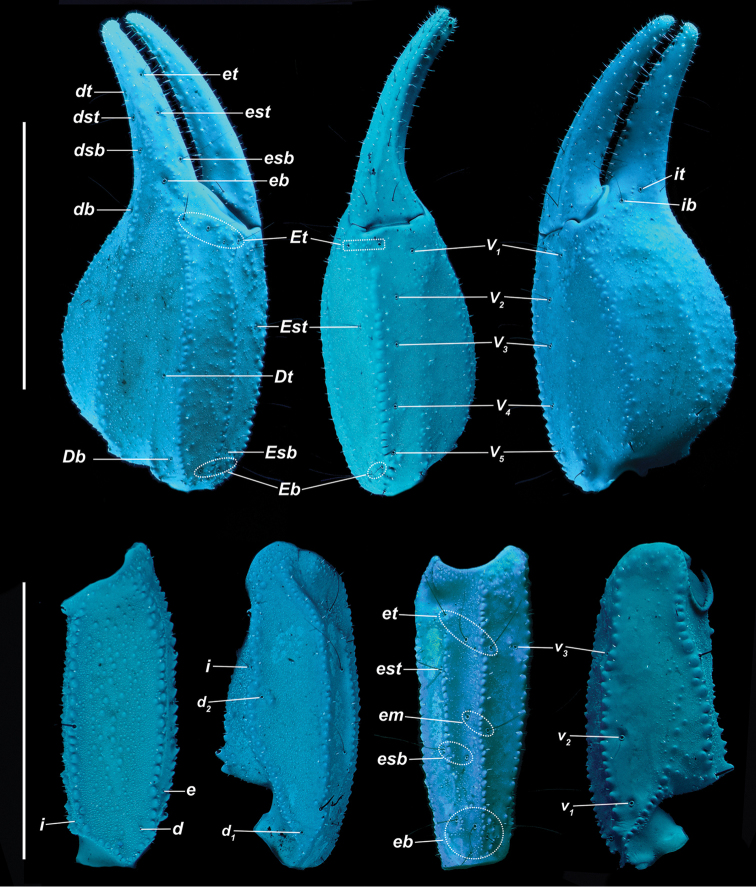
*Kovarikia oxy* sp. n. male holotype. Trichobothrial pattern. Scale bar = 5 mm; top bar for chela, bottom applies to femur and patella.

**Figure 10.**
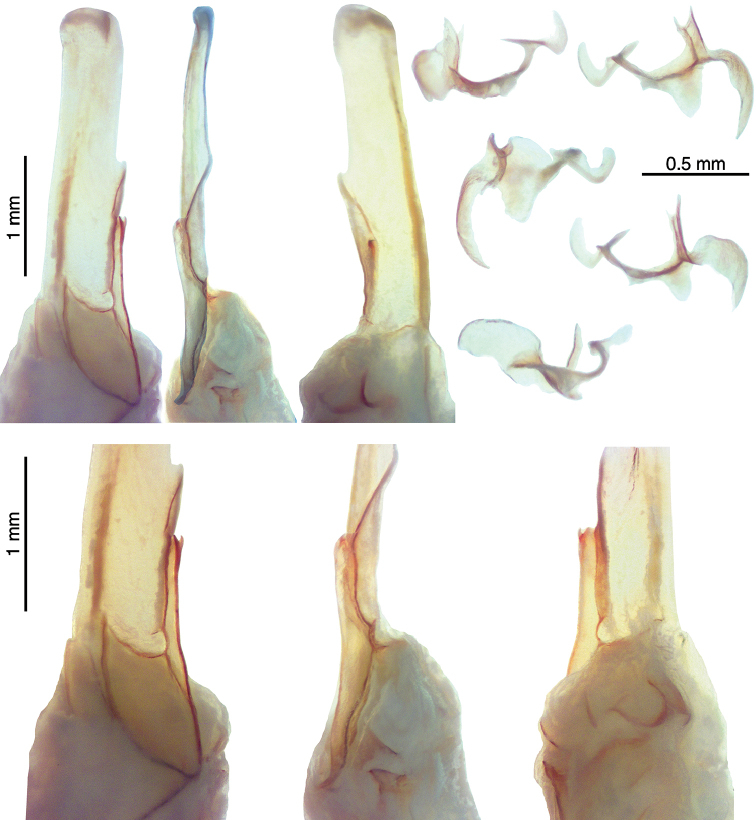
*Kovarikia oxy* sp. n., male holotype. Right hemispermatophore and mating plug (submerged in alcohol). **Upper-Left** Hemispermatophore median area and lamina, dorsal, internal, and ventroexternal views **Lower** Closeup of the median area and lamellar hooks, dorsal, internal, and ventral views. Note, embedded mating plug is visible in ventral view **Upper-Right** Mating plug, three dorsal views (left) and two ventral views (right).

Figs 8 , 9 , 10 ; Table 4

####### Type material.

*United States*: *California*: *Los Angeles Co*: male holotype (DMNS ZA.38178), Eaton Canyon Falls, San Gabriel Mountains. 34.19665°N, 118.10210°W, 475 m. 15 May 2014. R.W. Bryson Jr. and E. Zarza. Paratypes: Same locality. 15 May 2014. R.W. Bryson Jr. and E. Zarza, 2 ♂, 4 ♀ (DMNS ZA.38179–ZA.38184).

####### Etymology.

The specific name is a noun in apposition in reference to Occidental College, commonly referred to as Oxy, which lies at the base of the San Gabriel Mountains near Eaton Canyon, the type locality.

####### Diagnosis.

Large sized species for the family, with males up to 51.0 mm and females reaching 52.0 mm; pectinal tooth counts 12 for males and 11–13 for females. The species possesses the characteristics of genus *Kovarikia*: i.e. neobothriotaxy on ventral surface of chela, secondary lamellar hook on spermatophore, large crescent-shaped barb with a smooth edge on the mating plug, secondary exteromedian (*EM_c_*) carina on pedipalp patella. The holotype differs from the *K. savaryi* sp. n. holotype in the following: median eyes protrude well above carapace surface (only slightly above in *K. savaryi*); median carinal pair on sternite VII obsolete (essentially obsolete except for a few scattered small granules in *K. savaryi*); strongly granular intermediary carinae on metasomal segment I, the posterior 1/4 on segment II and posterior 1/5 of segment III (moderately granular on segment I, the posterior 1/5 of segment II, and posterior 1/6 of segment III in *K. savaryi*); lateral carinae on metasomal segment V crenulate and connecting with dorsolateral carinae at posterior 1/4 of segment (posterior 1/3 in *K. savaryi*); internal surface of femur with a few large granules arranged in a line along proximal 1/3 (scattered granules of various size, mostly on proximal 1/2 in *K. savaryi*); basitarsus retroventral setae count of 4/4:7/7:7/7:8/7 (4/4:5/5:5/5:7/6 in *K. oxy*). Differs from the other *Kovarikia* spp. by pectine counts and morphology of the chelal fingers and telson, as outlined below in the “Key to Species of *Kovarikia*”.

####### Description of holotype.

*Color* (Fig. 8): Carapace, trochanter, femur, patella, tergites, and metasoma have a brown base color with dark brown to black markings along the carinae of the pedipalp and metasoma. Legs are yellow brown with dark brown carinae. Pedipalp chelae are brown in color with darker reddish-brown coloration at the anterior portion of the palm where the fixed finger and movable finger meet. Chelicerae are light yellow with dark reddish-brown dentition. Vesicle portion of the telson is yellow-orange proximally, fading to cream on the distal third, with a dark reddish-brown to black aculeus. Pectines and genital operculum are light yellow to cream colored. *Morphology*: Carapace: trapezoidal with strongly emarginated anterior margin; surface with scattered granules; moderate median furrow traverses length of carapace; ratio of location of median eyes location (from anterior edge)/carapace length = 0.350; median eyes protrude well above carapace surface. Tergites: surface with small granules on distal 1/3–2/3 of tergites III–VI; tergite VII with two pairs of granular lateral carinae, and a moderate median hump. Sternites: III–VI smooth to very finely granular and without carinae; VII with granular ventral lateral carinae on posterior 2/3, median carinal pair obsolete (smooth). Spiracles: slightly ellipsoid and with median side rotated 30° away from posterior sternite margin. Genital Operculum: sclerites separated on posterior 1/5 exposing conspicuous genital papillae. Pectines: tooth count 12/12; middle lamellae 7/7; sensorial areas present on all pectine teeth. Metasoma: ratio of segment I length/width 1.02; segment II length/width 1.30; segment III length/width 1.54; segment IV length/width 2.07; segment V length/width 3.14. Segments I–IV: dorsal carinae are moderately denticulate on segments I–IV and have slightly enlarged distal denticles; dorsolateral carinae are moderately denticulate on segments I–IV with slightly enlarged posterior denticles; ventrolateral carinae are moderately crenulate on segments I–IV; strongly granular intermediary carinae occur on segment I, the posterior 1/4 on segment II and posterior 1/5 of segment III; ventromedian carinae are crenulate on segments I–IV; ventrolateral setae 2/2:2/2:2/2:2/2; ventral submedian setae 2/2:3/3:3/3:3/3. Segment V: dorsolateral carinae crenulate; lateral carinae crenulate and connecting with dorsolateral carinae at posterior 1/4 of segment; ventrolateral carinae crenulate; ventromedian carinae crenulate; intercarinal spaces with sparsely scattered granules; dorsolateral setation 2/2; lateral setation 2/2; ventrolateral setation 4/4; ventromedian setation 4/4. Telson: smooth to slightly granular with no subaculear tubercule and lacking LAS; posterior end of vesicle inflated toward the aculeus slightly forming a pair of smooth ventral grooves and carinae that stop at aculeus base; vesicle length/width 1.77; vesicle length/depth 2.30; vesicle length/aculeus length 2.66. Chelicerae: dorsal edge of fixed finger with four teeth, one distal, one subdistal, one median, and one basal, the latter two denticles formed as a bicuspid; ventral edge smooth; dorsal edge of movable finger has five teeth total comprised of one distal, two subdistal, one median tooth, and one basal tooth; ventral edge smooth; serrula with approximately 34 tines. Pedipalps (Fig. 9): trichobothrial pattern type C, neobothriotaxic: trichobothria *ib-it* positioned on very base of fixed finger, distance between positions of *Dt* and *Est* is less than that of *Dt* from palm base, *Db* dorsal of digital carina, *Et1* is clearly closer to the movable finger than V1, five ventral trichobothria (*V1*–*V5*); ratio of chela length/width 3.37; femur length/width 3.03; patella length/width 2.51; fixed finger length/carapace length 0.67. Chela: median denticles (MD) of fixed finger aligned and divided into six subrows by five outer denticles (OD); flanked by six inner denticles (ID); movable finger with six subrows of MD, five OD and seven ID. Chela carinae: Digital carina strong and crenulate; subdigital carina strong and crenulate but obsolete on distal 7/8; dorsosecondary moderate and crenulate; dorsomarginal very rounded, with large scattered granules; dorsointernal obsolete; interomedian weak, rounded with scattered granules; external carina moderate and crenulate; ventroexternal strong and crenulate; ventromedian moderate and crenulate on proximal 1/5, fading to smooth granules and essentially obsolete on distal 3/5; ventrointernal moderate to weak, rounded, with small scattered granules. Femur: dorsointernal and ventrointernal strong, black in color, and crenate; dorsoexternal carinae crenulate, brown to burgundy in color; ventroexternal reddish orange with scattered granules of various size; internal surface has a few large granules arranged in a line along proximal 1/3. Patella: dorsointernal and dorsoexternal carinae are crenulate and dark brown to burgundy in color; ventral internal and ventroexternal carinae crenate and dark brown to burgundy; external median carinae dark brown to burgundy and crenulate; seconday external median carina strong and on proximal 3/4, obsolete on distal 1/4, dark brown to burgundy and crenulate; internal surface has a large spur flanked by a few large granules. Legs: Ventral surface of telotarsi with single median row of 16–23 spinules terminating distally with two pairs of spinules. Two rows of small spinules occur on all basitarsi, fading proximally, but are very weak on basitarsus IV. Basitarsus populated with large irregularly placed darkly pigmented setae as follows: vental setae 5/5:6/6:6/6:6/6, retroventral setae 4/4:7/7:7/7:8/7, retromedian accessory setae 2/2:2/3:3/3:3/3, retrosuperior setae 2/2:2/2:2/2:2/2, and superior setae 3/3:3/3:3/3:3/3 (excluding DSM and DPS). Hemispermatophore (Fig. 10): Left hemispermatophore is 5.5 mm in length; lamina length 3.3, primary lamellar hook length 1.5, secondary lamellar hook length 0.5 (distance between tips of primary and secondary hooks), and trough difference (vertical distance between ventral and dorsal troughs) 1.0. Lamellar edges roughly parallel; terminus blunted with a very slight distal crest on the dorsal side. Primary lamellar hook extends somewhat from lamina base, is distinctly bifurcated, and is formed entirely from the dorsal trough. Secondary lamellar hook not bifurcated. A sclerotized mating plug with large asymmetric barb with a smooth edge was extracted from the ventrointernal aspect of the hemispermatophore median area.

**Table 4.** Measurements (in mm) of the type series of *Kovarikia oxy* sp. n.

	Male holotype (DMNS ZA.38178)	Male paratype 1 (DMNS ZA.38179)	Male paratype 2 (DMNS ZA.38180)	Female paratype 1 (DMNS ZA.38181)	Female paratype 2 (DMNS ZA.38182)	Female paratype 3 (DMNS ZA.38183)	Female paratype 4 (DMNS ZA.38184)
Total L	45	51	47	41	42.5	52	43
Cara L	5.35	6.2	5.8	5.75	6	6.1	6.1
Meso L	14.7	15.2	13.2	10.8	10.5	17.5	10.5
Met I L	2.5	3	2.7	2.4	2.5	2.65	2.6
Met I W	2.45	2.95	2.55	2.45	2.5	2.7	2.6
Met II L	3	3.5	3.3	2.9	2.95	3.2	3.1
Met II W	2.3	2.9	2.55	2.35	2.35	2.5	2.5
Met III L	3.55	3.95	3.7	3.2	3.35	3.6	3.4
Met III W	2.3	2.8	2.4	2.2	2.3	2.45	2.35
Met IV L	4.45	4.85	4.7	4	4.15	4.4	4.3
Met IV W	2.15	2.7	2.25	2.1	2.1	2.3	2.2
Met V L	6.6	7.6	7.1	6.2	6.3	6.8	6.6
Met V W	2.1	2.5	2.2	2.05	2.15	2.2	2.15
Tel L	5.75	6.55	6.25	5.8	6	6.25	na
Ves L	4.25	4.9	4.6	4	4.25	4.5	4.35
Ves W	2.4	2.7	2.6	2.25	2.45	2.5	2.55
Ves D	1.85	2.2	2	1.75	2	2.05	2
Acu L	1.6	1.7	1.65	-	1.75	1.8	
Fem L	5	5.75	5.5	5.1	5.35	5.55	5.5
Fem W	1.65	1.95	1.7	1.7	1.9	2	1.85
Pat L	4.9	5.65	5.2	5	5.35	5.55	5.4
Pat W	1.95	2.25	2.05	2.05	2.2	2.25	2.2
Chel L	9.1	10.4	10.05	9.4	9.85	10.35	10.1
Palm L	5	6.05	5.6	5	5.45	5.8	5.55
Palm W	2.7	3.1	3.15	2.8	2.85	3.05	2.85
Palm D	3.85	4.5	4.3	3.75	3.95	4.1	4.1
FF L	3.6	4.15	4.05	3.8	3.9	4.25	4.2
MF L	4.9	5.55	5.45	5.05	5.3	5.6	5.45
Pect Teeth	12/12	-/12	12/12	11/11	11/11	13/12	11/11

####### Measurements of male holotype

**(mm).** Total L, 45.0; carapace L, 5.35; mesosoma L, 14.70; metasoma L (additive without telson), 20.10; telson L, 5.75. Metasomal segments: I L/W, 2.50/2.45; II L/W, 3.00/2.30; III L/W, 3.55/2.30; IV L/W, 4.45/2.15; V L/W, 6.60/2.10. Telson: vesicle L/W/D, 4.25/2.40/1.85; aculeus L, 1.60. Pedipalps: femur L/W, 5.00/1.65; patella L/W, 4.90/1.95; chela L/W/D, 9.10/2.70/3.85; fixed finger L, 3.60; movable finger L, 4.90; palm L, 5.00.

####### Male and female variability.

Sexual dimorphism was evident in several morphological characters for *K. oxy*. Two-tailed Student’s t-tests indicated that the length of metasomal segment IV is significantly larger in males (*p* = 0.012) and that chelal palms are significantly wider (*p* = 0.019). One-tailed tests indicate that metasomal segment II is also longer in males (*p* = 0.048), telson vesicle widths are wider (*p* = 0.033), and that males have wider (*p* = 0.009) and deeper (*p* = 0.031) chelal palms. Larger sample sizes may reveal differences in additional characters, especially the lengths of the femur and metasomal segments I, III, and V.

### Key to species of *Kovarikia* Soleglad, Fet & Graham, 2014*

**Table:** 

1	Chelal fingers are relatively long when compared to the telson vesicle width: vesicle width / movable finger 0.35–0.41 (0.382) in the female and vesicle width / fixed finger 0.46–0.57 (0.510)	**2**
–	Chelal fingers are relatively short when compared to the telson vesicle width: vesicle width / movable finger length 0.43–0.50 (0.463) for the female and 0.43–0.51 (0.480) for the male, and vesicle width / fixed finger length 0.59–0.67 (0.619) for the female and 0.58–0.68 (0.642) for the male	**3**
2	Telson vesicular ridges are well developed and protrude beyond the aculeus juncture; telson vesicle is relatively wide, chelal palm depth / vesicle width 1.52–1.89 (1.680) for the female and 1.73–1.86 (1.803) for the male	***Kovarikia williamsi* (Gertsch & Soleglad, 1972)**
–	Telson vesicular ridges are of medium development and do not protrude beyond the aculeus juncture; telson vesicle is relatively thin, chelal palm depth / vesicle width 1.96–2.19 (2.052) for the female	***Kovarikia bogerti* (Gertsch & Soleglad, 1972)**
3	Pectinal tooth counts of male 12–13 and female 11–13	**4**
–	Pectinal tooth counts of male 9–11 and female 10–11	***Kovarikia angelena* (Gertsch & Soleglad, 1972)**
4	Telson vesicle is relatively long when compared to the chelal fingers, movable finger length / vesicle length 1.09–1.16 (1.115) for the female and 1.01–1.08 (1.047) for the male, and fixed finger length / vesicle length 0.80–0.86 (0.822) for the female and 0.74 (0.740) for the male	***Kovarikia savaryi* Bryson, Graham & Soleglad**
–	Telson vesicle is relatively short when compared to the chelal fingers, movable finger length / vesicle length 1.24–1.26 (1.252) for the female and 1.13–1.18 (1.157) for the male, and fixed finger / vesicle length 0.92–0.97 (0.944) for the female and 0.85–0.88 (0.858) for the male	***Kovarikia oxy* Bryson, Graham & Soleglad**
	* excluding male *K. bogerti*, unavailable at the time of study	

## Supplementary Material

XML Treatment for Kovarikia savaryi

XML Treatment for Kovarikia oxy

## Appendix 1

**Material examined**

*Kovarikia angelena*. *USA: California*: *Los Angeles Co*: Kanan-Duma Road above Malibu, Santa Monica Mountains. 34.05224, -118.79741, 378 m. 18 May 2014. R.W. Bryson Jr. and E. Zarza. 1 ♂ 3 ♀. *Ventura Co*: Yerba Buena Road near intersection with Hwy 1, Santa Monica Mountains. 34.07145, -118.95732, 134 m. 27 September 2013. R. W. Bryson Jr. 3 ♂.

*Kovarikia bogerti*. *USA: California*: *San Bernardino Co*: near Mountain Home Village, San Bernardino Mountains. 34.10895, -116.99125, 1244 m. 2 June 2013. R. W. Bryson Jr. 1 ♂ 1 ♀. *Riverside Co*: Hwy 74 near Mountain Center, San Jacinto Mountains. 33.70707, -116.75529, 987 m. 24 May 2014. D. Wood and C. Rochester. 4 ♀.

*Kovarikia oxy*. *USA: California*: *Los Angeles Co*: Eaton Canyon Falls, San Gabriel Mountains. 34.19665°N, 118.10210°W, 475 m. 15 May 2014. R.W. Bryson Jr. and E. Zarza. 3 ♂, 4 ♀.

*Kovarikia savaryi*. *USA: California*: *Orange Co*: Trabuco Creek Road near the entrance to Holy Jim Canyon, Santa Ana Mountains. 33.67699°N, 117.51733°W, 527 m. 15 April 2015. R.W. Bryson. 2 ♂, 5 ♀. *Orange Co*: Silverado Canyon Road, Santa Ana Mountains. 33.74614, -117.59327, 524 m. 16 April 2015, R.W. Bryson. 1 ♂.

*Kovarikia williamsi*. *USA: California*: *San Diego Co*: Barrett Flume. 32.62023, -116.72719, 449 m. 18 February 2014. D. Wood and D. Stokes. 1 ♂. *San Diego Co*: Escondido. 33.222830, -117.156470, 340 m. 10 March 2014. D. Wood and D. Stokes. 2 ♀. *San Diego Co*: Indian Valley Road, ca. 7 mi from junction with Hwy 79. 33.34891, -116.65568, 1176 m. 14 April 2014. D. Wood and D. Stokes. 1 ♀. *San Diego Co*: Mission Trails Regional Park. 32.82059, -117.06362, 80 m. 7 February 2014. D. Wood, R. Wood, and S. Wood. 1 ♂ 3 ♀. *San Diego Co*: Santa Ysabel Ecological Reserve, near USGS, San Diego Field Station Array 15. 33.13501, -116.65312, 1045 m. 6 March 2014. D. Wood and D. Stokes. 1 ♀. Same locality. 16 April 2015. D. Wood and D. Stokes. 1 ♀. *San Diego Co*: Mission Gorge. 32.81177, -117.07151, 34 m. 2010. M.R. Graham. 1 ♂, 1 ♀.

## Appendix 2

**Table:** Morphological variation among female *Kovarikia* measured for this study. For each character, the mean ± standard deviation are provided, with ranges in parentheses. All measurements are in mm.

	*K. angelena*	*K. bogerti*	*K. williamsi*	*K. oxy*	*K. savaryi*
	(N = 3)	(N = 4)	(N = 6)	(N = 4)	(N = 5)
Total L	44.67±5.80 (38.8–50.0)	49.38±1.89 (47.5–51.0)	48.92±1.59 (47.0–50.5)	44.63±4.99 (41.0–43.0)	52.30±4.89 (47.0–57.0)
Cara L	5.83±0.67 (5.10–6.40)	6.66±0.13 (6.50–6.80)	6.96±0.33 (6.40–7.30)	2.99±0.14 (5.75–6.10)	6.36±0.55 (5.75–7.20)
Met I L	2.65±0.25 (2.40–2.90)	2.70±0.22 (2.45–2.95)	2.88±0.13 (2.70–3.00)	2.54±0.10 (2.40–2.65)	2.93±0.23 (2.65–3.20)
Met I W	2.55±0.28 (2.30–2.85)	2.75±0.04 (2.70–2.80)	2.85±0.11 (2.70–3.05)	2.56±0.10 (2.45–2.70)	2.70±0.15 (2.55–2.95)
Met II L	3.13±0.25 (2.90–3.40)	3.29±0.11 (3.15–3.40)	3.41±0.20 (3.15–3.60)	3.04±0.12 (2.90–3.20)	3.52±0.27 (3.25–3.90)
Met II W	2.40±0.20 (2.20–2.60)	2.65±0.06 (2.60–2.70)	2.68±0.15 (2.50–2.95)	2.43±0.08 (2.35–2.50)	2.95±0.83 (2.50–4.45)
Met III L	3.32±0.18 (3.15–3.50)	3.65±0.13 (3.50–3.80)	3.73±0.23 (3.45–4.00)	3.39±0.14 (3.20–3.60)	3.78±0.28 (3.50–4.20)
Met III W	2.30±0.20 (2.10–2.50)	2.55±0.09 (2.45–2.65)	2.56±0.14 (2.40–2.80)	2.33±0.09 (2.20–2.45)	2.49±0.19 (2.30–2.80)
Met IV L	4.22±0.23 (4.00–4.45)	4.58±0.14 (4.40–4.75)	4.50±0.36 (4.00–4.95)	4.21±0.15 (4.00–4.40)	4.61±0.24 (4.35–4.95)
Met IV W	2.15±0.15 (2.00–2.30)	2.31±0.05 (2.25–2.35)	2.39±0.12 (2.25–2.60)	2.18±0.08 (2.10–2.30)	2.32±0.16 (2.20–2.60)
Met V L	6.67±0.50 (6.20–7.20)	6.84±0.23 (6.65–7.15)	7.27±0.44 (6.80–7.80)	4.28±0.18 (4.00–4.50)	7.36±0.62 (6.70–8.20)
Met V W	2.15±0.23 (1.90–2.35)	2.24±0.05 (2.20–2.30)	2.36±0.14 (2.20–2.55)	2.14±0.05 (2.05–2.20)	2.29±0.13 (2.15–2.50)
Ves L	4.57±0.18 (4.40–4.75)	4.40±0.15 (4.25–4.60)	4.79±0.54 (4.00–5.45)	4.28±0.18 (4.00–4.50)	5.03±0.47 (4.50–5.55)
Ves W	2.47±0.15 (2.30–2.60)	2.21±0.08 (2.15–2.30)	2.50±0.21 (2.20–2.80)	2.44±0.11 (2.25–2.55)	2.59±0.25 (2.35–3.00)
Ves D	2.03±0.16 (1.85–2.15)	1.94±0.05 (1.90–2.00)	2.11±0.22 (1.80–2.40)	1.95±0.12 (1.75–2.05)	2.10±0.14 (1.95–2.30)
Fem L	5.17±0.60 (4.50–5.65)	6.18±0.25 (6.00–6.55)	6.38±0.31 (5.90–6.70)	5.38±0.18 (5.10–5.55)	5.78±0.41 (5.35–6.40)
Fem W	2.18±0.63 (1.60–2.85)	2.19±0.03 (2.15–2.22)	2.24±0.09 (2.15–2.35)	1.86±0.11 (1.70–2.00)	2.01±0.15 (1.80–2.20)
Pat L	5.03±0.57 (4.40–5.50)	5.71±0.13 (5.60–5.90)	6.04±0.28 (5.60–6.40)	5.33±0.20 (5.10–5.55)	5.68±0.40 (5.30–6.30)
Pat W	2.18±0.28 (1.90–2.45)	2.53±0.12 (2.40–2.65)	2.60±0.14 (2.40–2.75)	2.18±0.08 (2.05–2.25)	2.37±0.13 (2.30–2.60)
Palm L	5.50±0.56 (4.90–6.00)	6.11±0.17 (6.00–6.35)	6.52±0.29 (6.10–6.80)	5.45±0.29 (5.00–5.80)	6.06±0.56 (5.50–6.80)
Palm W	3.50±0.88 (2.85–4.50)	3.09±0.10 (3.00–3.20)	3.29±0.28 (2.90–3.65)	2.89±0.10 (2.80–3.05)	2.37±0.13 (2.00–3.50)
Palm D	3.83±0.35 (3.50–4.20)	4.54±0.16 (4.40–4.70)	4.56±0.28 (4.15–4.90)	3.98±0.14 (3.75–4.10)	3.96±0.72 (2.80–4.75)
FF L	3.90±0.28 (3.60–4.15)	4.58±0.15 (4.40–4.70)	4.74±0.31 (4.30–5.10)	4.04±0.19 (3.80–4.25)	4.20±0.39 (3.75–4.75)
MF L	5.20±0.52 (4.60–5.50)	6.03±0.10 (5.95–6.15)	6.38±0.38 (5.80–6.75)	5.35±0.20 (5.05–5.60)	5.66±0.57 (5.05–6.45)
Pect Teeth	10.20±0.84 (9–11)	11.50±0.53 (11–12)	12.00±0.47 (11–13)	11.38±0.74 (11–13)	11.30±0.82 (10–13)

**Table:** Morphological variation among male *Kovarikia* measured for this study. For each character, the mean ± standard deviation are provided, with ranges in parentheses. All measurements are in mm.

	*K. angelena*	*K. williamsi*	*K. oxy*	*K. savaryi*
	(N = 4)	(N = 3)	(N = 3)	(N = 3)
Total L	43.45±1.94(41.30–46.00)	50.67±2.08(49.0–53.0)	47.67±3.06(45.0–51.0)	49.83±0.76(49.0–50.5)
Cara L	5.25±3.33(4.80–5.50)	7.05±0.25(6.80–7.30)	5.78±0.43(5.32–6.20)	6.18±0.33(5.85–6.50)
Met I L	5.12±5.07(2.42–2.70)	3.40±0.48(3.10–3.95)	2.73±0.25(2.50–3.00)	2.95±0.05(2.90–3.00)
Met I W	2.48±0.20(2.22–2.70)	3.05±0.13(2.95–3.20)	2.65±0.27(2.45–2.95)	2.82±0.19(2.60–2.95)
Met II L	3.08±0.21(2.80–3.30)	3.77±0.06(3.70–3.80)	3.27±0.25(3.00–3.50)	3.53±0.03(3.50–3.55)
Met II W	2.40±0.16(2.18–2.55)	2.95±0.18(2.80–3.15)	2.58±0.30(2.30–2.90)	2.75±0.18(2.55–2.90)
Met III L	3.30±0.22(3.00–3.50)	4.13±0.08(4.05–4.20)	3.73±0.20(3.55–3.95)	3.92±0.03(3.90–3.95)
Met III W	2.30±0.19(2.03–2.45)	2.87±0.18(2.70–3.05)	2.50±0.26(2.30–2.80)	2.68±0.18(2.50–2.85)
Met IV L	4.19±0.24(3.90–4.45)	4.90±0.62(4.20–5.40)	4.67±0.20(4.45–4.85)	4.92±0.10(4.80–5.00)
Met IV W	2.09±0.13(1.90–2.20)	2.67±0.18(2.50–2.85)	2.37±0.29(2.15–2.70)	2.48±0.18(2.30–2.65)
Met V L	6.41±0.41(5.95–6.80)	8.00±0.22(7.85–8.25)	7.10±0.50(6.60–7.60)	7.48±0.18(7.30–7.65)
Met V W	2.04±0.13(1.90–2.15)	2.65±0.13(2.50–2.75)	2.27±0.21(2.10–2.50)	2.38±0.13(2.25–2.50)
Ves L	4.18±0.32(3.90–4.50)	5.52±0.32(5.15–5.75)	4.58±0.33(2.40–2.70)	4.68±0.55(4.10–5.20)
Ves W	2.26±0.17(2.10–2.40)	2.87±0.15(2.70–3.00)	2.57±0.15(2.40–2.70)	2.67±0.12(2.60–2.80)
Ves D	1.81±0.24(1.55–2.10)	2.40±0.18(2.20–2.55)	2.02±0.18(1.85–2.20)	2.12±0.08(2.05–2.20)
Fem L	4.88±0.26(4.50–5.10)	6.70±0.33(6.40–7.05)	5.42±0.38(5.00–5.75)	5.50±0.10(5.40–5.60)
Fem W	1.69±0.10(1.55–1.80)	2.25±0.15(2.10–2.40)	1.78±0.16(1.65–1.95)	1.92±0.03(1.90–1.95)
Pat L	4.79±0.22(4.50–5.00)	6.22±0.30(5.90–6.50)	5.25±0.38(4.90–5.65)	5.35±0.13(5.25–5.50)
Pat W	1.98±0.14(1.82–2.15)	2.63±0.15(2.50–2.80)	2.08±0.15(1.95–2.25)	2.28±0.06(2.25–2.35)
Palm L	4.80±0.56(4.20–5.50)	6.93±0.40(6.50–7.30)	5.55±0.53(5.00–6.05)	5.75±0.18(5.55–5.90)
Palm W	2.66±0.22(2.45–2.90)	3.60±0.26(3.40–3.90)	2.98±0.25(2.70–3.15)	2.95±0.38(2.55–3.30)
Palm D	3.70±0.27(3.35–4.00)	5.17±0.25(4.90–5.40)	4.22±0.33(3.85–4.50)	4.43±0.23(4.20–4.65)
FF L	3.70±0.18(3.50–3.90)	5.02±0.38(4.65–5.40)	3.93±0.29(3.60–4.15)	4.38±0.72(3.85–5.20)
MF L	4.69±0.41(4.15–5.10)	6.67±0.60(6.10±7.30)	5.30±0.35(4.90–5.55)	5.30±0.18(5.15–5.50)
Pect Teeth	10.88±0.35(10–11)	12.83±0.41(12–13)	12.00±0.00(12)	12.33±0.52(12–13)
